# Therapeutic Drug Monitoring-Based Population Pharmacokinetics of Amikacin in Patients at a Teaching Hospital

**DOI:** 10.3390/antibiotics14060531

**Published:** 2025-05-22

**Authors:** Nadine Arnold Steffens, Estevan Sonego Zimmermann, Francine Johansson Azeredo, Rafael Linden, Luis Junior Finatto, Roberta Zilles Hahn, Alexandre Vargas Schwarzbold, Liliane Souto Pacheco, Natália Brucker

**Affiliations:** 1 Graduate Program in Pharmaceutical Sciences, Federal University of Santa Maria, Av. Roraima, 1000, Camobi, Santa Maria 97105-900, RS, Brazil; nadine.steffens@acad.ufsm.br; 2 Center for Pharmacometrics and Systems Pharmacology, University of Florida, Gainesville, FL 32827, USA; estevansz@hotmail.com (E.S.Z.);; 3 Laboratory of Analytical Toxicology, Feevale University, Novo Hamburgo 93525-075, RS, Brazil; rafael.linden@feevale.br (R.L.); betahahn@feevale.br (R.Z.H.); 4 University Hospital of Santa Maria, Federal University of Santa Maria, Santa Maria 97105-900, RS, Brazil; luisjfinatto@gmail.com (L.J.F.); alexvspoa@gmail.com (A.V.S.); lilispacheco@yahoo.com.br (L.S.P.)

**Keywords:** aminoglycosides, popPK, drug monitoring, antibiotics, Monolix

## Abstract

**Background**: Amikacin is still an essential antimicrobial to treat life-threatening infections, including multidrug-resistant microorganisms. The effectiveness of treatment has been correlated with the Cmax/MIC ratio, with a ratio of 8 being recommended, which is difficult to reach in some patients. Appropriate antibiotic exposure is important for knowing the disposition of the drug in the population. **Objectives**: We aimed to integrate therapeutic drug monitoring and a populational pharmacokinetic model to assess an optimal dose regimen and respective plasma exposure. **Methods**: Plasma levels of amikacin in peaks and troughs were determined by LC-MS/MS. The pharmacokinetic parameter was estimated to use nonlinear mixed effect modeling in Monolix^®^ software. The probability of target attainment was also determined using the Simulx™ software. **Results**: A total of 39 patients were enrolled. A one-compartment model with proportional error model best described amikacin pharmacokinetic parameters, providing a Cl of 1.49 L/h and Vc of 23.18 L. The model developed could characterize the pharmacokinetic profile in Brazilian patients who underwent therapeutic drug monitoring. **Conclusions**: Amikacin therapeutic drug monitoring should be associated with population pharmacokinetic analysis in dose optimization and individualization, helping maintain appropriate drug exposure in special populations such as critically ill patients. This strategy may contribute to enhancing clinical outcomes.

## 1. Introduction

Antibiotic resistance is a growing health problem, with multidrug-resistant pathogens (MDR) becoming more common annually in hospitals worldwide [1]. Hospital-acquired infections, sometimes related to MDR pathogens, are a significant cause of morbidity and mortality worldwide, being a challenge for public health [2,3]. Amikacin (AMK) is an aminoglycoside antibiotic used to treat life-threatening infections, being one of the options effective against Gram-negative and multidrug-resistant pathogens such as *Pseudomonas aeruginosa* and *Acinetobacter baumannii* [4,5].

Critical illness may affect the pharmacokinetics of drugs due to pathophysiological changes that occur in patients. These changes affect the organism’s behavior and lead to subtherapeutic concentrations or toxic effects. Achieving an ideal plasma concentration with the correct antibiotic is essential in treating infections [6,7,8,9].

Aminoglycosides, such as AMK, are concentration-dependent drugs and have bactericidal action through the irreversible inhibition of protein synthesis [10,11]. This antibiotic presents a narrow therapeutic index, with a pharmacokinetic/pharmacodynamic (PK/PD) target of peak concentration/minimum inhibitory concentration (Cmax/MIC) ratio ≥ 8 considered favorable for positive clinical outcomes [5,6,12]. Amikacin presents a risk of toxicity enrolled with exposure to high concentrations. Nephrotoxicity and ototoxicity are of significant concern, the first reversible after discontinuing the drug and the latter irreversible [12,13,14]. Trough concentrations ≤4 µg/mL, or ≤2.5 µg/mL, have been linked to lower odds of toxicity [6,15].

Therapeutic drug monitoring (TDM) can avoid such problems, helping adjust the dose and dosage intervals and ensuring patients achieve therapeutic concentration exposures [7,16,17,18]. TDM is defined as the measurement of drug concentration in the laboratory that, with appropriate interpretation, can aid in prescribing procedures and treatment compliance monitoring. When associated with pharmacokinetic analysis, whether populational pharmacokinetic (popPK) or PK/PD, it can support the individualization of therapy, improving dose strategies [19,20,21]. Recent studies suggest that higher-than-standard doses are required to reach adequate targeted concentrations for critically ill patients [9,12,22,23,24].

Without regard to the widespread use, limited data are available on AMK disposition in different patients, especially Brazilian patients. In this context, this study aimed to integrate therapeutic drug monitoring with the popPK model, capable of characterizing the pharmacokinetic profile of AMK in Brazilian patients. The optimal dose regimen and respective plasma exposure that may result in adequate exposure affecting life-threatening infections were also assessed.

## 2. Results

### 2.1. Patients

During the study period, 43 patients were enrolled. Four patients were removed from the dataset due to inappropriate collection time and lack of some important information. In total, 39 patients were included in the analysis. Demographic and clinical data from these subjects are described in Table 1. Most patients were male (*n* = 33, 84.61%). The mean age of the patients was 46.33 years, ranging from 4 to 75. *Klebsiella pneumoniae* (*n* = 20 cases) and *Pseudomonas aeruginosa* (*n* = 7 cases) were the most common microorganisms. Amikacin doses ranged from 225 mg to 1500 mg, administered every 12, 24, 48, or 72 h. The most common regimen was 1000 mg, administered every 24 h (*n* = 15, 35.7%). Six patients died during the hospital stay; it has not been assessed whether these deaths are related to the failure of treatment with amikacin.

Four patients had a body weight exceeding 100 kg, and fourteen patients exhibited a BMI greater than 25 kg/m^2^. Creatinine clearance (CrCl) demonstrated significant variability, with seven patients showing CrCl below 30 mL/min and ten patients recording CrCl above 120 mL/min.

A total of 113 amikacin concentrations were obtained, ranging from 2 to 6 samples per subject, 53 samples from the peak, and 60 samples from the trough. Mean ± SD amikacin blood concentrations were 41.96 ± 20.20 µg/mL and 8.75 ± 15.38 µg/mL for the peak and trough, respectively.

### 2.2. Amikacin popPK Model

First, different structural models were tested for all patients without covariates to the one- and two-compartment models. The result of this analysis suggested that the one-compartment model, with linear elimination and a combined 2 (additive + proportional) error model, presented a good description of the data. The basic parameters estimated for the population were total body clearance (Cl) and volume of distribution (Vd), with initial PK values of 2.04 L/h (RSE% 10.6) and 22.45 L (RSE% 11.5).

Subsequently, intra- and inter-individual random effects were explored in these populations. The data indicated that Ω in Cl (−266.82 decrease in the OFV) and Ω in Vd (−77.82 decrease in the OFV) were significant, improving the adjustments of the base model. The final estimates for the population PK parameters are summarized in Table 2. When analyzing the inclusion of some covariates like sex, age, body weight, creatinine clearance (CrCl), and body mass index (BMI), only CrCl improved the model, with a decrease in the OFV of −11.07.

The final model was fitted into 500 Bootstrap resampled datasets obtained from the original dataset. Bootstrap analysis was used to verify the stability and precision of parameter estimates of the final model, with results presented in Table 2.

### 2.3. Model Evaluation

**Figure 1:**
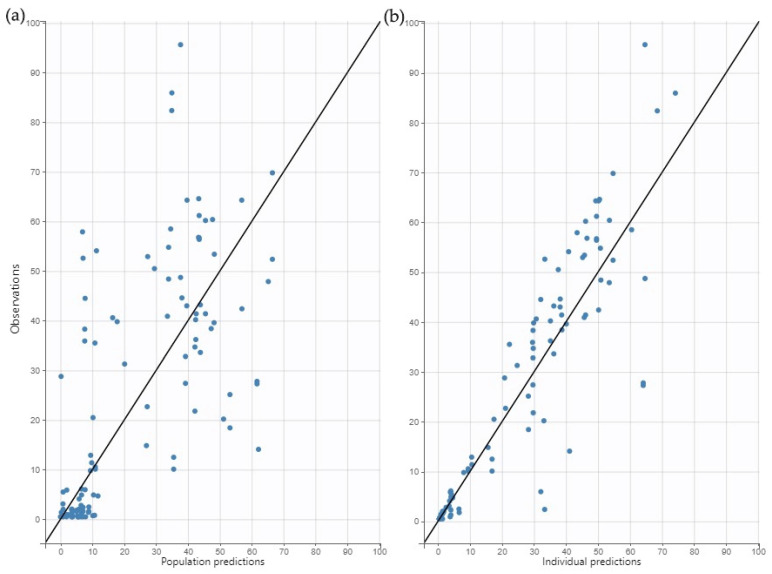
Goodness of fit plot (GOF): observed amikacin concentrations versus population prediction (**a**) and versus individual predictions (**b**). The black line represents the line of identity.

**Figure 2:**
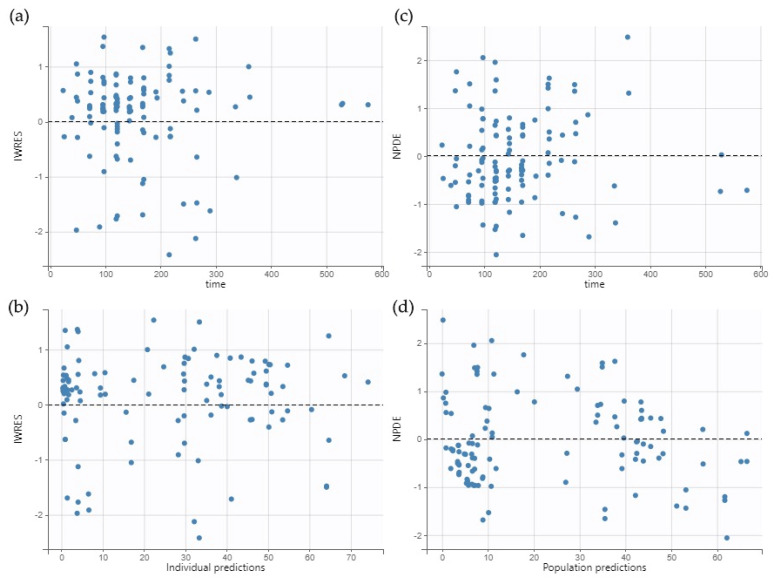
Individual weighted residuals (IWRES) versus time (**a**) and IWRES versus individual predictions (**b**). Normalized prediction distribution errors (NPDE) versus time (**c**) and NPDE versus population predictions (**d**). The black dashed line represents the zero-slope line.

The goodness-of-fit plots (GOF) were verified for model evaluation. Figure 1 shows the individual and populational observed versus predicted concentrations. Figure 2 shows the individual weighted residual (IWRES) versus time and individual predictions. Figure 2 also shows the Normalized prediction distribution errors (NPDE) versus time and population predictions. The plots revealed that the model presented no significant trend and fitted the data well.

**Figure 3:**
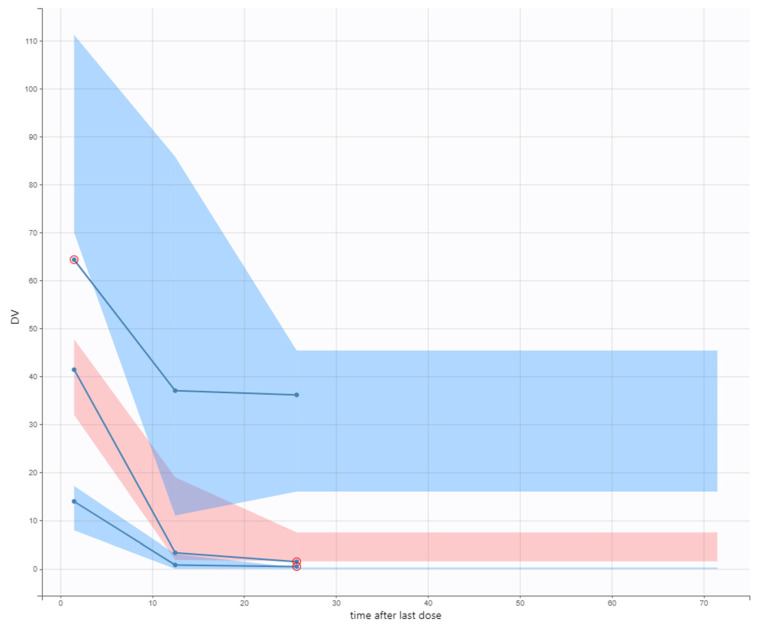
Visual Predictive Check (VPC) of amikacin plasma concentrations versus time after the last dose for the final model. Colored areas represent the prediction interval (theoretical percentiles) of simulated data from multiple Monte Carlo simulations. Blue areas correspond to the 10th and 90th percentiles, and the pale pink area corresponds to the 50th percentile. Blue lines inside the colored areas correspond to the 10th, 50th, and 90th empirical percentiles of the observed data. Red circles mark the outliers.

The Visual Predictive Check (VPC) plot (Figure 3) showed satisfactory predictive performance of the models, despite the large variability of the data and even though the variability in the percentiles was large. Still, IWRES and NPDE show that the model presents no tendency, confirming its suitability in fitting the data and being used to simulate new scenarios.

### 2.4. Dose Simulation and Target Attainment

**Figure 4:**
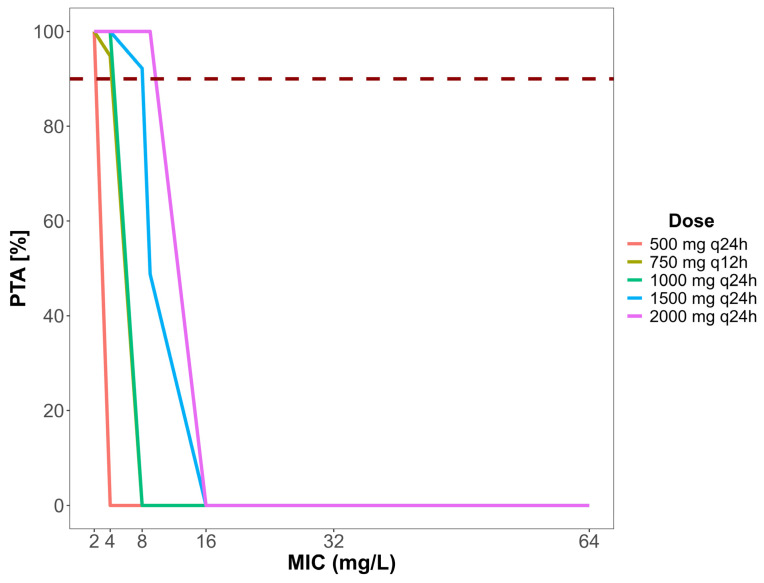
Achievement of the pharmacokinetic target (Cmax/MIC ≥ 8) according to the single-dosing day regimen administered through 1 h and the theoretical MIC of the strain in the Monte Carlo simulation. Dashed line represents the 90% probability of target attainment. The simulation was performed on 500 simulated patients. MIC, minimal inhibitory concentration.

After evaluating the model, based on the popPK parameters estimated, five dose regimens were simulated using Simulx™ version 2024R1. Based on expert recommendations for routine therapeutic drug monitoring (TDM) of AMK, we selected a target of achieving a Cmax/MIC ratio of ≥ 8 in at least 90% of 500 simulated patients. This is still the most adopted target in several studies [4,6,7,9,25,26]. Figure 4 presents the results of these simulations for different MIC values. The EUCAST MIC values for the pathogens most prevalent in our population were considered (*Pseudomonas aeruginosa* sensible MIC ≤ 16 mg/L and resistant MIC > 16 mg/L; *Klebsiella pneumoniae carbapenemase* susceptible MIC ≤ 8 mg/L and resistant MIC > 8 mg/L) [27].

Amikacin at 1000 mg was administered as a single infusion per day as part of the regimen and was sufficient to achieve the target, only until a MIC of 4 mg/L for all simulated patients. When aiming to treat infections with pathogens presenting an MIC greater than 9, even a 2000 mg single infusion per day regimen was insufficient to reach the plasmatic concentrations required.

Doses of 1500 mg achieved the target in 92.2% of patients simulated when evaluating a MIC of 8 mg/L. When adopting a 2000 mg dose administered as a 1 h infusion, adequate plasmatic concentrations could be reached to treat infections from *Enterobacterales* pathogens until a MIC ≤ 8 mg/L. When it comes to *Pseudomonas aeruginosa*, none of the doses simulated could reach adequate Cmax/MIC ≥ 8.

Given a trough target of Ctrough ≤ 2.5 µg/mL to prevent AMK nephrotoxicity, none of the simulated doses were effective.

## 3. Discussion

Amikacin is essential in antimicrobial treatments and is one of the weapons used to combat life-threatening infections. Intra and inter-individual PK variability due to pathophysiological changes in patients becomes complicated to accurately define the optimal dosage to be prescribed [28,29,30]. However, few studies assess the TDM profile of AMKBrazilian patients. In this present study, the popPK model, characterizing the pharmacokinetic profile of AMK in a Brazilian cohort of patients with different creatinine clearance who underwent non-routine TDM, was developed. We also evaluated the probability of different dose regimens to achieve a PK/PD target of Cmax/MIC ≥ 8 for clinically relevant pathogens.

According to the literature, several popPK models of AMK have been developed over the years, describing variabilities and significant differences in PK/PD in special populations such as elderly, critically ill patients and patients with cystic fibrosis, hematological diseases and burns, and some factors that may impact it, such as renal function, state of the disease, comorbidities, and others [10,12,22,31,32,33]. CrCl is a relevant covariate in studies involving popPK and PK/PD models for various antimicrobials, including aminoglycosides [8,25,28,29,34]. Creatinine clearance is a biomarker of glomerular filtration rate (GFR) estimated by the Cockcroft–Gault equation. In addition, there is a physiological relationship between drug concentrations eliminated almost entirely by the renal pathway and CrCl since this is a marker of renal dysfunction. [10,19,25,35]. In the present study, patients presented an AMK Cl of 1.49 L/h. Clearance values of AMK in other studies were higher, ranging from 2.25 L/h to 6.90 L/h [15,25,28,32,36,37]. Vd was estimated to be 23.18 L in our population. The literature describes Vd values ranging from 17.0 L to 41.5 L [10,25,28].

The PK parameters of Vd and Cl estimated in the present study are consistent with those reported by others previously published in the literature demonstrating that PK characteristics and properties of amikacin were adequately addressed [28,32,38]. Higher Vd values can be related to increased cardiac index, modifications in extracellular fluids, obesity, and intravenous fluids [5,6,9]. Aminoglycosides are almost eliminated by the renal pathway via glomerular filtration. Modifications in the renal function directly affect AMK elimination. When testing some covariates statistically significant to be included in the model, only CrCl was retained in the model, which also presents a biological plausibility [25]. It is important to note that weight and BMI, and age and creatinine clearance can be directly related, displaying a possible collinearity between these covariates [39]. It is important to emphasize that weight and BMI, and age and creatinine clearance are directly related. In our study, the vast majority of patients were in the same range of age and BMI, which does not allow us to extrapolate this collinearity.

VPCs show a good agreement between model prediction and experimental data in this study. However, the median and percentile bands are slightly oversized and reflect the variability of the data. The limited number of experimental points for each patient (only peak and trough) and the sample collection from our non-routine TDM practice are potential sources of variability in the present study. It is essential to highlight that residual plots indicate no trend, and residuals were normally distributed. These data support that PK parameters for AMK were correctly estimated, with standard error deviations below 30%. The GOF plots also present no tendency, supporting that the model was well-built. Nevertheless, it is necessary to perform a post-verification performance intended to guarantee the model’s validity in terms of clinical application and dose suggestions.

Therapeutic drug monitoring associated with popPK model analysis is a helpful tool to investigate new scenarios and dose regimens, enabling the planning of treatment strategies according to specific PK characteristics and comparing these different regimens, providing an individualized dosing approach. This strategy can help improve clinical outcomes while avoiding toxicity [15,28,33,37]. Implementing AMKs TDM in routine clinical practice can enhance treatment efficacy and prevent the escalation of bacterial resistance. This is achieved by enabling clinicians to appropriately adjust dosages based on the patient’s pharmacokinetic profile and the pharmacodynamic objectives of the therapy [40].

Pharmacokinetic/pharmacodynamic indices describe the relation of dose-concentration-effect of drugs in organisms. These PK/PD indices have been addressed in antibiotics therapies enrolling exposures with clinical efficacy and safety [7]. The success of the AMK treatment has been mainly associated with reaching two different targets: peak concentration/minimum inhibitory concentration (Cmax/MIC) ratio ≥ 8–10 or an area under the concentration–time curve (AUC) 80–120 mg. h/L. [6,7,25]. However, most studies applied Cmax/MIC as a clinical efficacy target [15,29,40,41,42].

Trough concentrations (Ctrough) are associated with toxicity, being commonly adopted as a target Ctrough ranging from ≤2.5 to ≤5.0 µg/mL [7,15,32,43]. We adopted the Ctrough ≤ 2.5 µg/mL as a target. In the evaluation of alternative dose regimens, ≥90% of patients reached toxic concentrations in simulations. Other studies demonstrated that around 30% of patients reached toxic concentrations higher than 4 µg/mL when evaluating similar dose regimens [4,5,32].

As sometimes patients can present a diminished clearance, increasing AMK concentrations, an alternative of extending the interval between the administrations, was previously mentioned [42]. On the other hand, patients with increased renal clearance may present lower AMK concentrations, requiring higher doses [15]. Pérez-Blanco et al. (2021) developed user-friendly and accessible AMK doses and nomogram web application tools, allowing easy evaluations of AMK initial dosage regimens based on primary patient and treatment information [5]. Also, we have some web sources that can estimate some available PK parameters.

Several amikacin dosage regimens have been studied by others aiming to reach a Cmax 64 µg/mL, corresponding to a Cmax/MIC ≥ 8 related to MIC breakpoints for *Enterobacteriaceae* and *Pseudomonas aeruginosa* by EUCAST [4,15,24,27,32]. Higher doses with a more extended administration interval have been an option suggested by many authors, especially for critical and elderly patients [10,24,32,41]. We only evaluated the probability of target attainment of some dose regimens previously addressed by other studies without making any dose suggestions. Results showed that the Cmax/MIC ≥ 8 ratio was achieved in ≥ 90% of patients with standard doses (1000 mg q24h–1500 mg q24h) until MICs of 8 mg/L or less. These data were consistent with the findings of Kato et al. (2017) [4].

For higher MIC values (greater than 9 mg/L), even 2000 mg administered as a 1 h infusion per day every 24 h did not reach a Cmax 64 µg/mL. A study conducted with Mexican patients with different renal functions showed similar results, where only with doses of 30 mg/kg or higher the target was almost 90% attained [25]. These results were also like the study described by Boidin et al. (2019) [15]. Several studies demonstrated that increased renal clearance might lead to therapeutic failure, especially of antibiotics eliminated by the renal pathway, leading to decreased plasma concentrations [25,41,44]. The Cmax/MIC target was most easily achieved in patients with reduced renal clearance, even in higher MICs, probably due to the reduced AMK Cl in this group, which makes high concentrations favorable even in standard regimens [45]. Thus, the higher the CrCl, the higher the AMK dose needed to reach optimal plasma concentrations.

There are some limitations to our study. First, the pharmacokinetic model construction was performed with data from TDM, which provided no information about the complete profile of blood concentrations. A second limitation is that the multiple collections of biological samples decrease the number of patient participants since they or the legally responsible need to accept and sign the informed consent form, which may have limited the identification of other significant covariates that could impact AMK’s PK. AMK’s PK profile can be described by one- and two-compartment models. Two-compartment PK profiles provide more PK parameters but are challenging to build adequately with samples from routine TDM; on the other hand, one-compartment is simpler, avoids overparameterization, and adequately describes samples from routine TDM.

## 4. Materials and Methods

### 4.1. Study Population

This is a single-center observational prospective study including critically and non-critically ill patients admitted to the Hospital Universitario de Santa Maria (HUSM), Brazil, between May 2018 and February 2020. Patients treated with AMK for several infections for at least three days were invited to participate in the study through an informed consent form. Exclusion criteria were burns, pregnancy, and those who refused to sign the informed consent. This study was approved by the Federal University of Santa Maria Ethics Committee (CAAE: 83200618.7.0000.5346).

### 4.2. Blood Samples and Amikacin Quantification

Blood samples were collected with EDTA anticoagulant to quantify AMK plasma concentrations: one sample was collected 30 min before drug administration (though). Another sample was collected 30 min after the end of the infusion time (peak). These samples were obtained after at least three days of therapy, at steady-state concentrations, considering an AMK half-life of approximately 2 h [19,46]. As AMK therapeutic drug monitoring (TDM) is not the routine analysis in HUSM, samples were centrifuged, and plasma was stored at −80 °C until the analysis. Plasma concentrations of AMK were determined after liquid–liquid extraction by a liquid chromatography–tandem mass spectrometry method (LC-MS/MS) previously published and validated, with a lower limit of quantification of 0.5 µg/mL. Results were expressed in µg/mL. The intra-day precision and accuracy ranged from 4.7 to 6.0% and 94.2 to 99.7%, respectively [47].

### 4.3. Data Collection

Patient medical records were individually reviewed using a standardized data collection model to collect information on therapy outcomes and adverse events. Clinical and demographic data were evaluated, such as sex, age, body weight, body mass index, diagnosis, comorbidities, biochemical and microbiological tests, and serum creatinine. Creatinine clearance was estimated according to the Cockcroft–Gault equation performed using the nephrological calculator from the website of the Brazilian Society of Nephrology [36,48]. In addition, therapy data such as dose regimen, infusion time, and the treatment period were evaluated. All data were used for the population pharmacokinetic analysis using Monolix software. Results were expressed as mean ± standard deviation (SD).

### 4.4. Population Pharmacokinetic (popPK) Analysis

#### 4.4.1. Model Construction and Evaluation

A population pharmacokinetic (popPK) model was developed using a nonlinear mixed effects modeling approach. The pharmacokinetic parameters of amikacin were estimated using MonolixSuite™ software version 2024R1 (Lixoft, Antony, France), based on individual plasma concentrations, using the stochastic approximation expectation-maximization (SAEM) algorithm. The model consists of two components: (1) a structural model that characterizes the concentration–time relationship and (2) a random-effects model that includes intra and interindividual variabilities and measurement errors in the pharmacokinetic (PK) parameters. To estimate the residual random errors of the model, which includes assay errors, model misspecification, and individual changes in the pharmacokinetic parameter, additive, proportional, and combined error models were evaluated.

The influence of some covariates such as age, weight, body mass index (BMI), and creatinine clearance (as continuous covariates) and sex and dialysis (as categorical covariates) on amikacin PK parameters was tested by a forward inclusion backward elimination stepwise method. A decrease in the objective function value (OFV) of at least 3.84 (*p* < 0.05) and 10.8 (*p* < 0.01) in the forward inclusion and backward elimination was required to keep the covariate in the model. The Akaike criterion (AIC) was also observed. Model comparison and selection of the final model were based upon numerical comparisons of OFVs, visual evaluation of basic goodness-of-fit plots, and Visual Predictive Checks (VPCs). Non-parametric bootstrapping of 500 datasets of a resampling procedure was also conducted to evaluate the stability and robustness of the final popPK model.

#### 4.4.2. Evaluation of Amikacin Doses by Monte Carlo Simulation

After defining the final model, the estimated distribution of amikacin PK parameters was used to simulate concentrations of five amikacin dosing regimens to determine the probability of reaching a Cmax/MIC target. The following dose regimens were simulated: 500 mg and 750 mg administered every 12 h, and 1000 mg, 1500 mg, and 2000 mg administered every 24 h as a 1 h infusion. For the simulation of these alternative scenarios, we used the Simulx™ software version 2024R1 (Lixoft, Antony, France). A Cmax/MIC ≥ 8 target, considering EUCAST MIC distribution of the most prevalent microorganisms, was adopted. A probability of ≥90% attainment was selected as our threshold for optimal treatment like other prominent authors do [4,9,10,15,27,42].

## 5. Conclusions

In conclusion, a popPK model capable of characterizing the pharmacokinetic profile of amikacin in Brazilian patients who underwent TDM was developed in this study, presenting a clearance of 1.49 L/h and a volume of distribution of 23.18 L. The PK/PD target of ≥90% of patients was achieved with standard doses only until MICs of 4 mg/L. With doses of 2000 mg/day or higher, it was possible to reach this target until a MIC of 8 µg/mL. Patients with decreased renal clearance can reach the PK/PD target most easily, even with standard doses. The model should further undergo predictive verification on many patients to ensure their predictive performance for clinical applicability, helping to maximize the likelihood of achieving efficacy and safety at AMK therapy. Routine AMK TDM associated with popPK models is a great tool in providing valuable information on AMK disposition. This tool can help define the most appropriate dose regimen, especially in populations such as the critically ill, and support appropriate drug exposure. This strategy could help improve clinical outcomes and develop more robust clinical protocols for antimicrobial therapy.

## Figures and Tables

**Table 1:** Clinical and demographic characteristics of patients enrolled in the study.

Parameter	Mean ± SD	Median [Range]
Male/Female	33/6	-
Age (Years)	46.33 (16.75)	51 [4–75]
Pediatric	2	-
Adult	28	-
Elderly	9	-
Weight (kg)	70.29 (25.07)	69.40 [15.60–143.80]
BMI (kg/m^2^)	24.57 (7.62)	23.20 [10.60–50.70]
Serum creatinine (mg/dL)	1.48 (1.55)	0.93 [0.16–8.35]
CrCl (mL/min) ^1^	100.3 (102.2)	79.01 [12.97–517.97]
Microorganisms isolated	*n*	
*Pseudomonas aeruginosa*	7	
*Acinetobacter baumannii*	1	
*Klebsiella pneeumoniae*	20	
*Escherichia coli*	2	
*Morganella morganii*	2	
*Enterobacter*	1	
*Staphylococcus haemophilus*	1	
*Enterococcus faecalis*	1	
*Vancomycin resistant Enterococcus*	1	
*Methicillin resistant Staphylococcus aureus*	1	

CrCl, creatinine clearance; BMI, body mass index; SD, standard deviation. ^1^ CrCl estimated according to the Cockroft-Gault formula.

**Table 2:** Final amikacin population pharmacokinetic parameters estimated with Monolix version 2024R1.

Parameter	Final Estimate	RSE (%)	Bootstrap Mean	Median	Difference (%)
Cl (L/h)	1.49	12.96	1.47	1.49	−1.29
Vd (L)	23.18	23.73	24.08	23.86	3.91
βCrCl on Cl	0.004	57.15	0.005	0.0041	24.93
*IIV* (%CV)					
ΩCl	0.67 (74.8)	17.01	0.63	0.62	−6.21
ΩVd	0.47 (49.89)	37.69	0.41	0.42	−12.51
Proportional error model	0.38	14.86	0.39	0.39	1.7

## Data Availability

The data that support the findings of this study are available from the corresponding author, N.B., upon request. Some data are unavailable due to privacy or ethical restrictions.
